# Brought to you courtesy of the red, white, and blue–pigments of nontuberculous mycobacteria

**DOI:** 10.3934/microbiol.2020026

**Published:** 2020-11-17

**Authors:** Tru Tran, Stephanie N. Dawrs, Grant J. Norton, Ravleen Virdi, Jennifer R. Honda

**Affiliations:** 1 College of Osteopathic Medicine, Lake Erie College of Osteopathic Medicine, Bradenton, Florida, USA; 2 Center for Genes, Environment, and Health; Department of Immunology and Genomic Research, National Jewish Health, Denver, Colorado, USA

**Keywords:** Nontuberculous mycobacteria, pigment, virulence, nonchromogens, photochromogens, scotochromogens

## Abstract

Pigments are chromophores naturally synthesized by animals, plants, and microorganisms, as well as produced synthetically for a wide variety of industries such as food, pharmaceuticals, and textiles. Bacteria produce various pigments including melanin, pyocyanin, bacteriochlorophyll, violacein, prodigiosin, and carotenoids that exert diverse biological activities as antioxidants and demonstrate anti-inflammatory, anti-cancer, and antimicrobial properties. Nontuberculous mycobacteria (NTM) include over 200 environmental and acid-fast species; some of which can cause opportunistic disease in humans. Early in the study of mycobacteriology, the vast majority of mycobacteria were not known to synthesize pigments, particularly NTM isolates of clinical significance such as the *Mycobacterium avium* complex (MAC) species. This paper reviews the overall understanding of microbial pigments, their applications, as well as highlights what is currently known about pigments produced by NTM, the circumstances that trigger their production, and their potential roles in NTM survival and virulence.

## 1. Introduction

Multicolored creatures comprise the macroscopic and microscopic worlds. Elucidating the evolutionary advantages for developing particular coloration and patterns has intrigued the scientific community for centuries. Examples include the cryptic coloration of the *Megascops asio* eastern screech owl, aposematism in *Phyllidia varicose* nudibranchs, advancement of sexual selection among *Paradisaea decora* Goldie's bird of paradise, and protection of human and animal skin from UV radiation through the production of melanin. For photosynthesizing plants, green chlorophyll is the most widely recognized and understood pigment. Likewise, pigments derived from microbes are well-studied and often used in coloring that is added to clothing/textiles, cosmetics, and food [1]. But, for the microbes from which they are derived, pigments are diverse chemicals produced in direct response to a broad range of conditional circumstances with multifunctional biological properties and activities.

## 2. Bacterial pigment varieties and their functions

Empirically, bacteria produce pigments to facilitate survival in the harsh terrestrial and marine environments from which they are typically associated, including air-water interfaces, glaciers, ice cores, soil, lava caves, salt lakes, groundwater, deep sea hydrothermal vents, and hot springs [2]. Independent of photosynthesis, the functional purposes of microbial pigments are to: *(i)* protect against UV radiation, oxidants, extreme temperature changes, and desiccation, *(ii)* act as antimicrobial agents, *(iii)* recover nutrients, *(iv)*, avert phagocytosis, *(v)* transport iron [2],[3] and *(vi)* promote bacterial pathogenicity and virulence (*e.g*., enhanced virulence of melanin-producing *Vibrio cholerae* by increasing cholera toxin production) [4]. In some circumstanaces, a variety of bacteria use quorum-sensing to produce pigments, including production of violacein by *Chromobacterium violaceum* that results from the coordinated behavior of the bacterial community [3],[5],[6].

Because molecular oxygen is required for pigment production, obligate anaerobes are typically devoid of pigment [7]. On the other hand, the aerobic Phylum *Actinobacteria* is composed, in part, of pigment-producing bacteria; of which *Streptomyces* produces a wide variety [8]. Among this phylum, certain species of acid-fast *Mycobacteria* are described as nonchromogenic in the dark, but become photochromogenic and produce bright orange/pink pigments upon short-wave light exposure, suggesting light influences pigment production [7]. Yet other bacteria produce a wide variety of pigments of varying colors including *Serratia* (red), *Vibrio* (red), *Halobacterium* (rose-pink), *Allochromatium* (orange-brown/pink-purple), and *Thiocapsa* (rosy-peach) [9].

## 3. Spectrum of bacterial pigments

**Figure 1.**
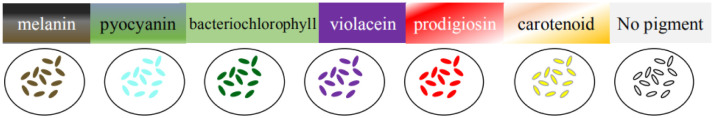
Spectrum of microbial pigments.

Bacterial pigments include melanin, pyocyanin, bacteriochlorophyll, violacein, and prodigiosin; of which, carotenoids are the most widely observed and studied [10] (Figure 1). Herein, we systematically discuss each pigment in terms of its biology, source, function, applications and implications, biosynthesis and regulation, and interaction with the immune system, if known.

### 3.1. Melanin

Biology: A negatively charged, hydrophobic, high molecular weight chromophore composed of polymerized phenolic or indolic, dark-colored natural pigments (*e.g*., black/brown eumelanins, red/yellow pheomelanins, and dark brown-black allomelanins). Source: Melanin is typically produced by organisms across many kingdoms as well as marine bacteria including *Vibrio cholerae* and *Proteus mirabilis* [11]. Function: Melanin is critical to the bacteria that synthesize it because it provides an innate shield against solar radiation, desiccation, toxic heavy metals, oxidative stress, and hyperosmotic shock [11]. For example, to survive extreme aquatic marine habitats, *Shewanella* uses melanin as a terminal electron acceptor during shifts between aerobic and anaerobic respiration, particularly when environmental oxygen is scarce [12]. Separately, *Legionella pneumophila* melanin is used to increase iron availability during iron-limiting conditions by reducing Fe^3+^ to bioavailable Fe^2+^ [13]. Application: Melanin has been developed to increase the sun protection factor in sunscreens, biocontrol, and in the industrial production of light-stable, environmental friendly insecticides [14]. Biosynthesis and regulation: Most of the bacterial melanins are formed through multi-process transformations of aromatic compounds involving the amino acids methionine and tyrosine using similar biochemical processes in humans. In bacteria such as *Sinorhizobium meliloti*, the *trxL* gene codes for thioredoxin, which stimulates tyrosinase activity needed for chromophore production [15]. Other bacteria including *Escherichia coli, Pseudomonas putida, Cornybacterium glutamicum, and Streptomyces griseus* synthesize melanin from malonyl-CoA catalyzed by polyketide synthases [14],[16]. In several species of *Streptomyces*, the polyketide synthase RppA responsible for melanin synthesis, is regulated by transcriptional regulator AdpA that also controls sporulation processes [15],[17],[18]. In addition to polyketide synthases, other diverse enzymes, substrates, and multiple pathways are used to regulate the synthesis of bacterial melanin such as temperature, nutritional factors (*e.g*., copper, zinc, nitrogen, and oxygen), or stress as observed for the seawater bacterium *Marinomonas mediterranea* [14]. Interaction with the immune system: Melanin increases bacterial virulence by reducing susceptibility to host defense mechanisms and by influencing the host immune responses to infection [11]. For example, *Burkholderia cepacia* melanin acts as an antioxidant, attenuating macrophage superoxide production [19]. To protect against oxidative stress, melanin from *Burkholderia cenocepacia* neutralizes reactive oxygen species (ROS) generated by the oxidative burst in host cells [20]. Melanin from *V. cholerae* increases ROS production, toxin and pilus expression, as well as enhances host colonization and protection from amoeba predation [4],[21]. During chronic infections, *Pseudomonas aeruginosa* increases melanin production to resist oxidative stress [14].

### 3.2. Pyocyanin

Biology and source: A zwitterion, water soluble pigment canonically produced by *P. aeruginosa* and is one of the most widely characterized bacterial virulence factors with antimicrobial and anti-inflammatory properties [22]. Jayaseelan *et al.*, provides a comprehensive review of pyocyanin biology [23]. Briefly, pyocyanin's most widely recognized characteristic is its secreted blue-green color, commonly observed in sputa from *P. aeruginosa*-infected patients [24]. Function: Pyocyanin shows oxidase activity and inhibits the growth of other competitors while easily penetrating biological membranes [25]. Application: Because of its distinct color, pyocyanin is used as a coloring agent for clothes made of cotton and linen and in food coloring [26]. It also has the capacity to degrade pesticides, and inhibits fungal growth by arresting the electron transport chain [27],[28]. Biosynthesis and regulation: A variety of studies have suggested cell-density dependent quorum sensing autoinducers control many of the known virulence factors of *P. aeruginosa*, including pyocyanin production [29]. Pyocyanin production is regulated by two copies of a seven-gene operon that synthsize phenazine-1-carboxylic acid which is converted to pyocyanin by two additional modifying enzymes, an adenosylmethionine dependent methyltransferase (*phzM*) and a flavin dependent hydroxylase (*phz*S) [30],[31]. The synthesis of pyocyanin by *P. aeruginosa* is also strongly dependent on natural nutrients such as carbohydrates, fats, proteins, peptones, magnesium chloride, and glycerol. In the absence of these factors, bacterial density and pyocyanin pigment concentration are greatly reduced [32],[33]. Interaction with the immune system: In the human airway, pyocyanin oxidizes glutathione and NADH resulting in increased ROS (*e.g.,* H_2_O_2_) [34], redox homeostasis disturbance, and epithelial cell injury and death [35]. Importantly, *P. aeruginosa* mutants lacking pyocyanin show attenuation in both acute and chronic mouse models of lung infection [25]. As a virulence factor, pyocyanin reduces bacterial clearance from the lungs by accelerating the clearance of neutrophils from inflamed sites via apoptosis [36].

### 3.3. *Bacteriochlorophyll* (BChl)

Biology: Pigments such a chlorophyll are employed by photosynthetic organisms such as plants and certain bacteria by harvesting energy from sunlight. There are several different chlorophyll pigments, each with different stereochemistry, esterifying alcohol, methylation, and light-harvesting efficiency due to their distinctive absorption characteristics. Source: Phototrophic bacteria (*e.g*., purple bacteria, green sulfur bacteria, *Heliobacteria*) produce the photosynthetic pigment BChl, which encompasses pigments BChla-BChlg to conduct photosynthesis, but does not generate oxygen as a byproduct [37]. Typically, BChl-producing phototrophic bacteria are facultative, obligate anaerobes or obligate aerobic bacteria [37]. There are two main groups of purple bacteria - those that produce BChl anaerobically in the light and dark (*e.g*., *Rhodobacter spharoides, Rhodobacter capsulatus, Rhodobacter rubrum*) and those that synthesize BChl under both anaerobic and aerobic conditions in the light (*e.g., Rhodovulum sulfidophilum, Roseobacter* sp., *Rubrivivax gelatinosus*) [38]. Function: As strict anaerobes, green sulfur bacteria perform phototrophic processes without the production of oxygen and use reduced sulfur compounds as electron donors. They also produce chlorosomes, specialized antenna complexes that contain high concentrations of BChl *c, d,* or *e* [39]. Application: Because of its photochemistry and light harvesting properties, BChl has been studied as a potential sensitizer in photodynamic therapy aimed to target tumor cells [40],[41]. Biosynthesis and regulation: Through numerous studies aimed to understand differences in BChl expression and production under varying environmental conditions, the biosynthetic pathways of BChl have been identified [37],[39],[42],[43]. However, the exact role of each individual BChl on bacterial metabolism and survival has yet to be elucidated. Interaction with the immune system: Currently, the role of BChl in modulating the host immune response in human is ill-defined.

### 3.4. Violacein

Biology and source: A water-soluble violet/purple pigment produced by diverse bacterial genera such as *Pseudoalteromonas, Cillimonas, Duganella*, and *Janthinobacterium*; of which, *Chromobacterium violaceum* is the most well-known [44]. Gram-negative *C. violaceum* is typically found in tropical water and soil and was first isolated from the Amazon River [45]. Function: Violacein provides protection from UV radiation and protozoal predation, shields the bacterial lipid membrane from peroxidation caused by hydroxyl radicals, and induces apoptosis of leukocyte cell lines [46]–[50]. Additionally, violacein demonstrates antibacterial activity against Gram-positive and Gram-negative bacteria, particularly *Staphylococcus aureus* [51],[52] and *Escherichia coli* (>50 mg/L) [51]. Violacein also exerts antimicrobial activity against protozoans and metazoans [49]. For example, *Acanthamoeba castellanii* exhibits markers of cell death including decreased feeding, cell rounding, and increased caspase-3-like activity with violacein exposure [53]. Application: It has been suggested that violacein demonstrates anti-cancer properties. For example, introducing violacein into colorectal cell line Caco-2 mediates production of ROS, resulting in damage to mitochondrial membranes, leakage of cytochrome c, and cell death via apoptosis as the result of caspase-3 activation [54]. Biosynthesis and regulation: The biosynthesis pathway of violacein begins with L-tryptophan as a substrate that is sequentially processed by five different enzymes encoded by five different genes: *vioA, vioB, vioC, vioD,* and *vioE* [55]. Interaction with the immune system: In the HL60 myeloid leukemia cancer cell line, violacein induced apoptosis at half maximal inhibitory concentration (700 nM), but this could not be recapitulated in other types of leukemia cells, human leukocytes, and monocytes [47]. The anti-cancer mechanism of violacein appears to mimic the activities of tumor necrosis factor α (TNF-α) in signaling cellular apoptosis through the activation of caspase 8, transcription of nuclear factor kappaB (NF-κB), and p38 mitogen-activated protein (MAP) kinase [47].

### 3.5. Prodigiosin

Biology and source: A light-sensitive, alcohol-soluble, red-pigment produced by various Gram-negative bacteria including *Serratia marcescens* and *Streptomyces* spp. Prodigiosin derivatives have also been identified in the marine γ-proteobacterium, *Pseudoalteromonas* [56]. Function: *Vibrio* producing prodigiosin show increased survival against UV exposure compared to non-prodigiosin-producing *Vibrio* isolates, suggesting a role of protection in the environment [57]. Similar to other pigments discussed, prodigiosin exerts antimicrobial activity. Purified prodigiosin from *Vibrio* demonstrates bacteriostatic properties against *Staphylococcus, Streptococcus,* and *Klebsiella,* although its mechanism of action remains poorly understood [58]. Prodigiosin also demonstrates activity against chloroquine-resistant strain *Plasmodium falciparum*, induces synthesis of autolysins in *Bacillus cereus,* and stimulates production of ROS that prevents formation of biofilm in *P. aeruginosa* [56],[59]. In the case of herpes simplex virus type 1 and 2, prodigiosin exerts antiviral activity by inhibiting NF-κB and Akt signaling pathways *in vitro* and *ex vivo* in cultured porcine corneas [60]. The pathway in which prodigiosin provides antiviral relief involves the inhibition of anti-apoptotic activity of TNF-α mediated by NF-κB [60]. Applications: Prodigiosin has been used in food coloration to replace the synthetic pigments used in the food industry, coloration of polyolefines, as well as applied in sunscreen to increase the sunscreen protection factor [61]. Biosynthesis and regulation: Biosynthesis of prodigiosin is regulated by PigP—a master transcriptional regulator of multiple genes involved in production of prodigiosin [62]. Mutations in *PigS*, a member of PigP regulon, resulted in reduced production of prodigiosin by decreasing the transcription of the biosynthetic operon [62]. *Pseudoalteromonas* also synthesizes other analogs of prodigiosin including cycloprodigiosin (cPrG) and 2-(p-hydroxybenzyl) prodigiosin (HBPG). The key difference in structure between prodigiosin and *c*PrG is the cyclization between the C-3 pentyl group and C-4 on pyrrole ring C of 2-methyl-3-n-amyl-pyrrole. HBPG differs in structure to prodigiosin by the para-addition of phenol to C-10 of the pyrrole ring A of 4-methoxy-2,2′-bipyrrole-5-carbaldehyde [56]. Interaction with the immune system: Prodigiosin shows immunosuppressive properties by inhibiting both T-cell receptor dependent and independent proliferation of T-cells [3],[63]. Additionally, *S. marcescens* prodigiosin induces apoptosis in hematopoietic cancer and human colon cancer cell lines [64]. Other novel activities attributed to prodigiosin include negatively altering biofilm integrity of *P. aeruginosa* and H_2_O_2_ and hydroxyl radicals stimulated by prodigiosin show strong cleaving properties towards DNA and RNA, but no effect on protein [65]. *Pseudoalteromonas* cPrG was shown induce apoptosis in several cancers including acute human T-cell leukemia, acute promyelocytic leukemia, human and rat hepatocellular cancer, human breast cancer, and TNF-stimulated human cervix carcinoma [56],[61].

### 3.6. Carotenoid

Biology and source: Lipid soluble, yellow-orange-red-pink [66] colored organic, natural pigments widely observed in bacteria, but also found in plants, algae, and fungi [67]. The carotenoid pigments typified for bacteria include orange-pink canthaxanthin, echinenone pigments of *Micrococcus roseus*, red astaxanthin pigments of marine bacteria such as *Halobacterium* spp., and the orange-staphyloxanathin membrane bound pigments of *S. aureus* [68]. Function: Bacterial carotenoids shield against UV radiation and oxidative damage and facilitate membrane fluidity that promote viability at low temperatures and facilitate nutrient transport [69],[70]. *S. aureus* carotenoids are necessary and sufficient to promote its pathogenicity. Specifically, loss of staphyloxanathin significantly decreases the virulence of *S. aureus* in murine skin abscesses or systemic models of infection and its production by group A *Streptococcus* results in large skin lesions in mice [71]. Carotenoids are extremely hydrophobic and are used to stabilize bacterial membranes while being described as antioxidant agents [9]. For example, xanthomodanin exhibits antioxidant properties by inhibiting photodynamic lipid peroxidation in liposomes to protect against photodamage [72]. Carotenoids of Antarctic heterotrophic bacteria facilitate adaptation to harsh and cold environments by modulating their membrane fluidity under a wide range of temperatures [73]. Application: Carotenoids are used in food products in solution or suspension in vegetable oils and to color margarine [9]. As a potential biologic in medicine, the carotenoids derived from the soil-dwelling *Kocuria rhizophila* and *Corynebacterium glutamicum* demonstrate antimicrobial activity against the nematode *Caenorhabditis elegans* [74]. *Holomonas sp*.-derived carotenoids demonstrate broad antimicrobial activities against *Klebsiella, Pseudomonas*, and *Staphylococcus*, but also anti-cancer properties [75]. Biosynthesis and regulation: Carotenoid synthesis begins with a 5-carbon building block isopentenyl pyrophosphate followed by chain elongation catalyzed by prenyl transferases that synthesize geranyl, farnesyl, or geranylgeranyl pyrophosphate–all of which are precursors of carotenoids [76]. Interaction with the immune system: Carotenoids have been demonstrated to play several key immune functions such as stimulating production of immunoglobulin production by B cells, as well as increasing the sensitivity and activity of CD4+ helper T cells, CD8+ cytotoxic T cells, and natural killer cells against viruses, bacteria, parasites, and fungi [77]. In addition evidence suggests dietery β-carotene prevents bladder, kidney, ear, and gut infections in rats with vitamin A deficiency, and reduced ear infections in children [77]. Carotenoids also exhibit anti-cancer effects on various cancer cell lines such as esophagus, breast, liver, and cervix [78].

## 4. Environmental factors that influence bacterial pigment production

Summarized above are the varieties and biological summaries of six well-described bacterial pigments. Stimulation of pigment production is driven by a myriad of factors including nutitional factors and varying environmental conditions as highlighted in Table 1.

**Table 1.** Environmental factors that influence pigment production.

Environmental factor	Promote or inhibit pigment production	Genus, species	Reference
**Nutritional factors**			
Nutrient-rich conditions	Promote	*Flexibacter elegans*	[79]
High availability of phosphate	Inhibition of fluorescent pigment	*Flexibacter elegans*	[79]
Trace sulfate	Promote	*Flexibacter elegans*	[79]
Methanol as a carbon source	Promote pink pigment production	*Acinetobacter wofii,* *Methylobacterium extorquens*	[80] [66]
Succinate as a carbon source	Promote yellow-green fluorescent pigment	*Pseudomonas fluorescens*	[81]
Presence of copper	Promote prodigiosin	*Serratia marcescens*	[65]
Low iron	Promote carotenoids	*Synechococcus* species	[82]
Nicotine	Inhibit carotenoids	*Mycobacterium marinum*	[83]
**Environmental conditions**			
High acidity	Inhibition of fluorescent pigment	*Flexibacter elegans*	[79]
	Promote carotenoids	*Mycobacterium smegmatis*	[84]
	Production of carotenoids	*Mycobacterium tuberculosis*	[85]
Hyperosmotic stress	Production of melanin	*Vibrio cholera*	[86]
Nutrient-rich conditions	Promote	*Flexibacter elegans*	[79]
High availability of phosphate	Inhibition of fluorescent pigment	*Flexibacter elegans*	[79]

## 5. Spotlighting the current knowledge of mycobacterial pigments

Presently, the *Mycobacterium* genus consists of over 200 recognized species [87], of which *Mycobacterium tuberculosis* (tuberculosis) and *Mycobacterium leprae* (leprosy) are the most widely recognized. However, the majority of the genus is comprised of nontuberculous mycobacteria (NTM), of which, a subset can cause environmentally acquired opportunistic pulmonary and skin disease of varying severity in susceptible individuals. The majority of the NTM do not produce pigment; however, some are known to synthesize yellow, orange, or rust colored colonies [88]. Whether the pigment is visible by the naked eye or by visualization under a microscope depends on its concentration within an organism [89].

Of the bacterial pigments discussed earlier in this review, carotenoids are the most well-characterized pigments of NTM, functioning as free radical scavengers to protect against oxidative stress [89]. Using thin-layer chromatography, Tarnok *et al*. elucidated the carotenoids of *Mycobacterium phlei* (xanthophyls)*, M. avium, M. kansasii* (alpha and beta-carotene and lycopene)*, M. intracellulare, M. aurum, M. marinum, M. gordonae,* and *M. scrofulcaceum* [89],[90]. Carotenoid gene regulation in NTM has also been studied in some detail. The *crt* gene cluster has been shown to encode for enzymes responsible for the synthesis of *crtB, crtE, crtl,* and *crtY* carotenes in mycobacteria. When the *crtB* gene for yellow pigment production is transferred via cosmid from photochromogenic *M. marinum* into nonchromogenic *M. smegmatis*, yellow *M. smegmatis* colonies are produced [91]. However, *M. smegmatis* possesses other genes such as *sigF* which is also involved in biosynthesis of carotenoids [92]. Interestingly, the *sigF* sequences in *M. smegmatis* are highly similar to *M. tuberculosis*, which canonically regulate the structure and function of the mycobacterial cell wall [92]. *sigF* mutant knockouts are more sensitive to H_2_O_2_, oxidative stress, and show decreased transformation efficiency [92],[93]. But besides its biological effects against oxidative stress, the role of carotenoids in mycobacterial virulence remains ill-defined.

In 1959, Ernest Runyon introduced the Runyon classification scheme to classify *Mycobacteria* based on their rate of growth, colony morphology, and production of pigment in the presence or absence of light [94]. Besides the nonchromogenic category which include the *M. avium* complex (‘MAC’, such as *M. avium, M. intracellulare, M. chimaera*), *M. terrae* complex, *M. ulcerans, M. xenopi, M. simiae, M. malmoense, M. asiaticum, M. haemophilum*, NTM are also classified as photochromogens (colorless colonies in the dark, but synthesize pigments upon light exposure) and include *M. kansasii, M. marinum, M. szulgai* or scotochromogens (produce pigments in both light and dark conditions) such as *M. gordonae* and *M. scrofulaceum*. Today, the Runyon classification system is considered to be outdated due to advances in mycobacteriology [95], but also because some bacteria previously thought to be nonchromogens actually express some levels of pigmentation. For example, the MAC species were originally classified as non-pigmented, but MAC isolates from acquired immunodeficiency syndrome (AIDS) patients were later discovered to synthesize pigments [96].

MAC consists of 12 validly published species [97] including *M. chimaera, M. avium*, and *M. intracellulare* and are typically classified as nonchromogenic, showing non-pigmented colonies on Middlebrook 7H10 agar plates. MAC species typify slow-growing mycobacteria (SGM) with culture times > 14 days and are readily recovered by microbiological culture from soil and freshwater systems [98]. Currently, there is little existing knowledge regarding the biology and significance of pigment among the mycobacteria. Of interest, *Methylobacterium* sp. are Gram-negative α-proteobacteria, pink-pigmented facultative methylotrophs that use methanol as an energy source. Similar to NTM, *Methylobacterium* sp. also inhabit water distribution systems and form biofilms and produce pink carotenoids (*e.g*., *Methylobacterium extorquens*) [66]. Studies have suggested that *Methylobacterium* sp. is an indicator for the presence or absence of NTM [99] and *Methylobacterium* sp. inhibit the development of *M. abscessus, M. chelonae*, and *M. fortuitum* biofilms [100]. Whether the relationship between nonchromogenic NTM and other pink environmental bacteria such as *Methylobacterium* sp. is fortuitous or detrimental remains to be elucidated.

To understand the role and significance of pigments in NTM biology and the reasons and circumstances for mycobacterial pigment production, we highlight and revisit existing knowledge on this topic below.

## 6. Factors that promote the production of mycobacterial pigments

In 1938, sunlight, UV light, and ambient light from a 100-watt lamp were factors found to influence pigment production among unidentified pathogenic acid-fast organism found in the tropical platyfish (*Platypoecilus masculatus*) [7]. Acid-fast cultures incubated in the dark were colorless, whereas those incubated in presence of sunlight or UV light developed a deep orange color. Furthermore, data obtained from the same study suggested shorter light rays induced higher production of pigments in these organisms whereas full coloration resulted after 15 minutes exposure to sunlight, 30 minutes with ambient light, and one minute with UV light. It is noteworthy that prolonged exposure was lethal as demonstrated by cessation of pigmentation and unobtainable subculture post exposure [7]. Baker *et al*. went on to show that of 185 acid-fast isolates tested, 24 (13%) produced pigment when exposed to light [7]. Of the 24 isolates, two (8%) were colorless when grown in the dark that became light orange when exposed to light, of which, one was identified as ‘*M. avium* type strain.’ The remaining 22 isolates (92%) produced modest light orange when cultured in the dark and brilliant orange pigment in diffuse daylight, suggesting UV light exposure is an inducer of pigment. UV-mediated production of pigment is also dependent on bacterial viability, as only live *M. marinum* synthesized pigments upon UV exposure; dead *M. marinum* remained non-pigmented [7]. To protect from UV radiation, mycobacteria including *M. tuberculosis, M. fortuitum, M. avium, M. intracellulare, M. marinum, M. kansasii, M. smegmatis,* and *M. flavescens* show an inverse correlation between UV sensitivity and the presence of carotenes; that is, the higher the concentration of carotene present, the less sensitive the mycobacterium to UV exposure [101].

### 6.1. Storage time, length of incubation, and incubation temperature

In 1970, Gordon and Pang reported an isolate of *M. fortuitum*, originally described as non-pigmented, appeared to produce a black soluble pigment after 10 years of storage at 4 °C, indicating that extended time in storage facilitates pigment production [102]. Additionally, extended incubation time can also drive pigment production. RGM *M. goodii* can cause post-traumatic wound infections including osteomyelitis, producing smooth to mucoid, off-white to cream colonies in 2–4 days that turn yellow-orange after 10–15 days of incubation [103]. Smooth SGM *M. colombiense* produces yellow pigments with increased age (after a 4–5 weeks of incubation) [104]. Of interest, rough *M. colombiense* isolates lacking pigment exhibited impaired sliding motility, biofilm formation, and production of glycopeptidolipids, reducing the organism's ability to survive and invade host cells [104]. The brown-black pigment produced by *M. magertense* isolated from blood cultures from patients with prosthetic valve endocarditis was shown to be driven by incubation temperature. At 42 °C, *M. magertense* did not produce pigments. However, at 35 °C light-brown pigmented colonies appeared and even darker brown pigmented colonies were observed after seven days of incubation at 30 °C [105].

### 6.2. Media used for culture

Pigment production by mycobacteria may also be driven by the type of culture media used for colony observation. In 1975, the pigment intensity of *M. fortuitum* was described for three microbiological culture media: (1) Middlebrook 7H10, (2) Lowenstein-Jensen (L–J), and (3) American Trudeau Society (ATS) culture media [106]. At day 3 post-inoculation, no growth was observed after incubation at 25 °C, but buff colored *M. fortuitum* colonies were noted after incubation at 36 °C on all three media types. By day 21, rust, brown, and tan colonies with scattering of brown colonies were respectively observed on 7H10, L–J, and ATS plates incubated at 25 °C. Plates that were incubated at 36 °C showed darker brown and intense brown colonies on 7H10 and L–J plates while the same tan/brown colonies were observed on ATS plates. Although outdated now, this study demonstrated two key points. Of the three media types, darker pigmented *M. fortuitum* colonies were more frequently observed for L–J media compared to 7H10 and ATS media and pigmented colonies can darken with higher incubation temperature and prolonged incubation time.

### 6.3. Immunodeficiency and susceptibility to antibiotics

A significant percentage of patients with AIDS in the 1980's showed fatal infections caused by disseminated, nonchromogenic *M. avium* and *M. intracellulare* infection [96]. In contrast to same strains recovered from patients without AIDS, *M. avium* and *M. intracellulare* from AIDS patients showed deep, yellow carotenoid pigment producing colonies. These pigmented variants were also less tolerant to antibiotics such as β-lactams, more hydrophobic, and showed faster growth rates compared to non-pigmented variants [107]. Yet another study demonstrated that MAC isolated from 30 patients with acquired immune deficiency syndrome (86%) produced a deep yellow pigmented colonies, which remained susceptible to clofazimine, cycloserine, and ansacmycin, but resistant to isoniazid, streptomycin, ethambutol, ethionamide, and rifampin [96].

### 6.4. Acid exposure

NTM are found in exogenous and endogenous environments where exposure to acid fluctuates greatly. For example, NTM thrive in environments rich in humic and fluvic acids, acidic soil, and acidic brown water swamps [108]–[110]. NTM also resist exposure to the acidic condition of the stomach [111] and replicate in airway epithelial cells under pH 4 and 2 conditions [112]. Additionally, pH ranges between 4.5 to 6.5 are encountered by NTM the phagolysosomes of infected macrophages [84].

To study the role of acid exposure on the production of pigments by NTM *in vitro*, *M. smegmatis* was gradually induced to produce pigments by acidifying Sauton's media cultures to pH 6.0 [113]. As a result, the bacteria accumulated a dark brown fluorescent pigment noticeable in both cells and the culture medium. By studying the pigment's absorption spectra, a class of porphyrins involved in heme metabolism were identified that also served as antioxidants, providing protection against ROS. In another study, *M. abscessus, M. fortuitum, M. smegmatis, M. chelonae, M. goodii*, *M. intracellulare,* and *M. avium* were patched onto agar media of varying pH [84]. At pH 7, no pigments were observed from any of the tested species. At pH 6, yellow-orange pigment was reported for *M. smegmatis* and *M. goodii*. At pH 5.5, *M. intracellulare, M. avium*, *M. abscessus*, and *M. chelonae* produced pigments while pigmentation was maintained in *M. smegmatis* and *M. goodii*. Only two species were tested at pH 5, of which only *M. abscessus* continued to produce yellow-orange pigment but not *M. chelonae*, suggesting acid exposures modulate pigment production. Finally, *M. avium* subsp. *paratuberculosis* (MAP) is responsible for paratuberculosis in ruminants and is a potential pathogen associated with inflammatory bowel diseases in humans [114]. MAP consists of two main types, S (sheep) and C (cattle) (91). Of the two types, type S is well-known to produce yellow pigments, but not type C. However, carotenoid production for MAP type C was stimulated when the culture was grown aerobically in the media with pH below 5.5 [115].

In conclusions, the primary drivers for mycobacterial pigment production described to date are tallied in Table 2.

**Table 2.** Circumstances that promote pigment production in NTM.

Circumstance	Date of discovery	Example	Reference
Light wavelength	1937	*M. avium* type strain	[7]
Extended time in storage at 4 °C	1970	*M. fortuitum*	[102]
Extended incubation time	1999	*M. goodii*	[103]
Incubation temperature	2015	*M. magertense*	[105]
Media used for culture	1976	*M. fortuitum*	[106]
AIDS	1985	*MAC*	[96]
Acidifying culture media	2016	*M. smegmatis*	[113]

## 7. Conclusion

Bacterial pigments were first discovered and discussed more than 100 years ago; yet, their roles in mycobacterial virulence are undisputedly uncharacterized. Mycobacterial pigments are associated with photoreception and survival; but their synthesis, regulation, and physiological role have not been studied at large. In fact, most studies performed are now considered antiquated and outdated. However, the possibility that NTM may acquire pigment producing genes from other typically pigmented non-NTM bacteria within a shared environment may be a new area of exploration. Understanding the role for pigments in NTM biology could potentially open up new research opportunities that might lead to new therapeutic interventions. As an example, one of the enzymatic steps involved staphyloxanthin (a carotenoid pigment and virulence factor of *S. aureus* against ROS from host cells) synthesis has been found to resemble those for cholesterol synthesis. Thus, cholesterol-lowering drugs such as statins have been applied to therapeutically decrease production and activity of this virulence factor leading to increased killing of *S. aureus* by host cells [116]. Perhaps similar strategies may be applicable for targeted chemotherapy against NTM, providing alternative new therapies against NTM infections that are difficult to treat. Until then, we advocate for more studies to elucidate the role of pigments in the biology of mycobacterial organisms.
